# Discrepancies with Final Diagnosis in Head and Neck Intraoperative Frozen Section

**DOI:** 10.1007/s12105-026-01959-3

**Published:** 2026-08-14

**Authors:** Scott Albright, Alexandra Isaacson, Robert Robinson

**Affiliations:** Department of Pathology, University of Iowa, Iowa City, USA

**Keywords:** Frozen section, Head and neck, Final diagnosis discrepancy

## Abstract

**Purpose:**

Creating a frozen section slide and crafting an interpretation is a multistep process with multiple possible points of failure. Head and neck frozen section pathology presents particular technical and diagnostic challenges. The aim of our study was to identify discrepancies between head/neck intraoperative and final diagnoses, determine the site of process failure, and assess the potential causes.

**Methods:**

Concordance data from head and neck intraoperative and final diagnoses (2020–2023) were queried from our LIS-embedded quality assurance system. Discrepancies were categorized as major and minor, with major defined as those that would have altered the surgical procedure.

**Results:**

We found 71 total discrepancies out of 1844 head and neck cases (3.9%). Major discrepancies accounted for 48/1844 (2.7%). Only major errors were considered for further study and were classified primarily into three categories: (1) inadequate gross sampling by the prosector, usually involving mucosal tissues of excised large specimens for evaluation of margins for dysplasia/carcinoma (14/48 cases; 29%), (2) insufficient sectioning into the block leaving undetected tissue unexamined (9/48 cases; 19%), and (3) interpretative errors (23/48 cases; 48%). The most common major interpretative errors involved the diagnosis of squamous dysplasia (9 cases), failure to identify minimally invasive squamous carcinoma (4 cases), failure to identify parathyroid and thyroid tissues (5 cases), and incorrect diagnoses of non-squamous neoplasms (5 cases). Two errors (4%) were ascribed to processing artifact issues in the frozen section.

**Conclusion:**

Half of diagnostic discrepancies occurred at steps prior to microscopic interpretation. Management of specimen grossing and tissue selection in large specimens, adequate block sectioning, and adherence to diagnostic criteria for difficult but common issues such as squamous dysplasia, minimally invasive carcinoma and thyroid/parathyroid morphology may help reduce intraoperative/final diagnosis discrepancies.

## Introduction

Frozen section analysis serves as a cornerstone of intraoperative pathology consultation, guiding surgical decision-making in real time. Interpreting frozen sections in the head and neck region can be notoriously difficult with numerous diagnostic pitfalls, lookalikes, and complex anatomy that complicate the diagnostic process. The technical challenges of generating a frozen section slide and limited turnaround time also contribute to the high-stress environment.

The most common means of identifying discrepancies in frozen section interpretation is by comparison of the frozen section results with the permanent section diagnoses, usually rendered on formalin-fixed, paraffin-embedded tissue slides, along with any additional gross pathology examination and tissue blocks. Identifying these discrepancies is an important part of quality assurance in surgical pathology. Studies have demonstrated major frozen section error rates ranging from 1 to 4% for general surgical frozen sections overall and for head and neck frozen sections specifically [1–3]. 

The study goals were to define, enumerate and classify head and neck frozen section discrepancies with a specific focus on the sites and causes of process failure.

## Methods

### Review Process and Categorization of Errors

A retrospective review of head and neck frozen section concordance data was conducted from the University of Iowa Health Care laboratory information system (LIS)-embedded quality assurance system from January 2020 through December 2023. 1844 head and neck cases were identified from this time period which required intraoperative frozen section assessment.

In this department’s LIS, frozen section quality assurance data is recorded by the pathologist rendering the final diagnosis. This data includes whether the pathologist agreed or disagreed with the reported frozen section results. Any discrepancy for that case is then classified as a major or minor disagreement as well as by type of error: sampling, interpretive, or technical. Major discrepancies are recorded in the final report with documentation of a clinician alert.

We retrospectively reviewed all major and minor discrepancies identified through the LIS for the time period listed. Cases were reclassified as major or minor by an expert head and neck pathologist. The type of error was also reevaluated based on thorough review of the case slides.

### Discrepancy Categorization

#### Major Discrepancies by Type of Error

Sampling errors during gross examination were defined by cases with a discrepancy between frozen section results and a subsequent block submitted only for permanent sections, in which the latter contained the diagnostic focus.

Block cutting errors were defined by cases where the diagnostic focus remained in the block and was not viewed in the frozen section slides, but was identified in subsequent sections of the frozen block remnants submitted for permanent evaluation.

Interpretive errors were defined by histologic misinterpretation of the frozen section slides on re-review at final case sign out. These interpretative errors were further subclassified by diagnostic category: squamous cell carcinomas (SCC), high grade dysplasia, non-squamous lesions, thyroid/parathyroid tissue, and metastases.

Technical errors included those discrepancies related to issues in the handling, cutting, or staining of the frozen section tissue.

Errors were classified as “minor” in cases where the difference in diagnosis would not have changed the outcome of the procedure. Examples included SCC versus suspicious for SCC or SCC at margin vs. SCC < 1 mm from margin, based on the surgeons’ usual intraoperative actions in response to these diagnoses at our institution.

#### Protocol for Cutting Frozen Sections

Our institutional standard practice is to cut two levels on all frozen section blocks. For head and neck margins which are oriented perpendicular to the margin, at least two sections are cut. For shaved (en face) margins submitted parallel to the true margin, at least three sections are cut. Additional sections are cut as needed or appropriate.

#### Practice Settings of the Frozen Section Service

Our surgical pathology group operates within a subspecialty model with several pathologists covering each subspecialty service. The frozen section service is covered in a general manner by all the surgical pathology faculty.

## Results

Over a four- year period (2020–2023), the frozen section laboratory processed 7441 individual frozen sections which included 1844 (25%) head and neck cases. The total discrepancy rate in head and neck frozen sections was 3.9% (71/1844), with a major discrepancy rate of 2.6% (48/1844) (See Table 1).

**Table 1:** Laboratory frozen sections from 2020 through 2023

	Total lab frozen sections	Total head and neck frozen sections	Total head and neck discrepancies	Head and neck major discrepancies	Head and neck minor discrepancies
2020	1790	440	21	15	6
2021	2089	502	14	8	6
2022	1837	460	19	13	6
2023	1725	442	17	12	5
Total	7441	1844	71	48	23

Discrepancies related to gross sampling occurred in 14 cases (29%). These errors most frequently occurred in cases involving invasive squamous cell carcinoma and high-grade dysplasia (11 cases). Of these 11 cases, 10 of them were discrepancies related to margin assessment. Three gross sampling errors occurred in cases with non-squamous lesions. These included a Warthin tumor, a papillary thyroid carcinoma (PTC), and an inverted Schneiderian papilloma.

**Fig. 1:**
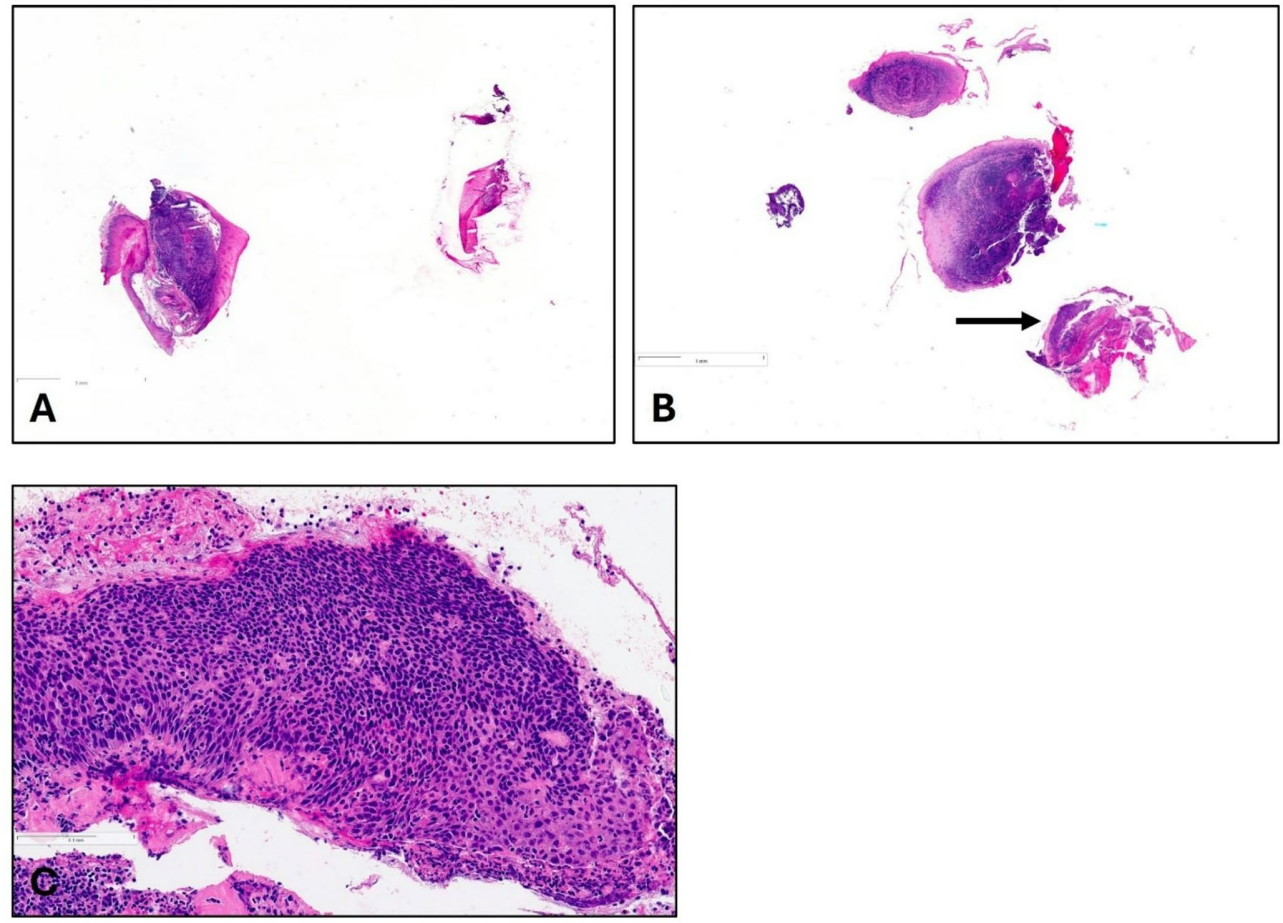
Insufficient sectioning into frozen block. **A** (1X) Frozen section margin with two pieces of tissue on the slide. Reported as “Negative for in situ or invasive carcinoma.” **B** (1X) Permanent section with three pieces of tissue on the slide. Final diagnosis of “At least squamous cell carcinoma in situ.” **C** (20X) Permanent section with final diagnosis of “At least squamous cell carcinoma in situ.”

Block cutting errors during frozen sectioning resulted in a major discrepancy in 9 cases (18%). In these cases, diagnostic material in the block was not present on the frozen section slide but was identified after deeper sectioning of the block for permanent section (See Fig. 1). The errors due to frozen block cutting were spread evenly between the diagnostic categories (Table 2).

**Table 2:** Major discrepancies by type of error and diagnostic category from 2020 through 2023

	Inadequate gross sampling	Insufficient sectioning into frozen block	Interpretative error	Technical errors
High grade dysplasia/CIS	7	2	9	1
Invasive squamous cell carcinoma	4	2	4	
Parathyroid/thyroid	0	1	5	
Non-squamous neoplasms	3	1	3	1
Metastases	0	3	2	
Total	14	9	23	2

**Fig. 2:**
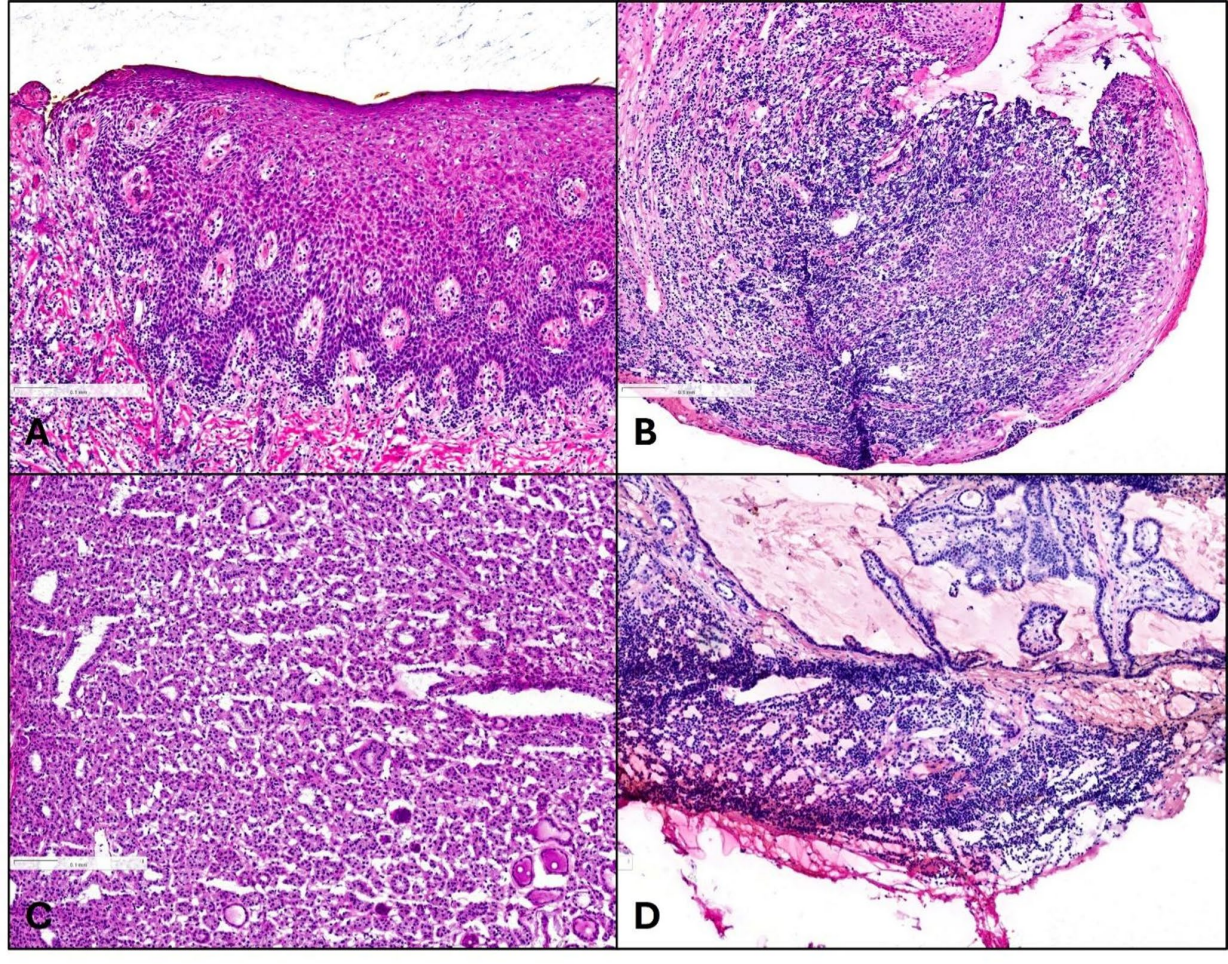
Examples of frozen sections with major interpretive discrepancies. **A** (10X) Benign reactive squamous epithelium of hard palate margin called “squamous cell carcinoma in situ” at frozen. **B** (10X) Frozen section with 0.5 cm focus of nonkeratinizing squamous cell carcinoma called benign glossotonsillar sulcus. **C** (10X) Frozen section of benign thyroid parenchyma reported as hypercellular parathyroid tissue at frozen. **D** (10X) Neck lymph node submitted as ‘thyroid versus parathyroid’. The lymph node contained metastatic papillary thyroid carcinoma but was diagnosed as benign thyroid tissue at frozen section

Interpretive errors accounted for 23 major discrepancies (48%) (Fig. 2). Misdiagnosis of squamous dysplasia resulted in the most common major interpretative error (9 cases). Of these nine cases, six were originally reported as “negative for high grade dysplasia” but were overread as “positive for high grade dysplasia” by the final pathologist. Three cases were interpreted as “positive for high grade dysplasia” at frozen section, but were subsequently interpreted as negative by the final pathologist.

Identification of focal invasive squamous carcinoma (4 cases) and distinction between parathyroid and thyroid tissue (5 cases) were other frequent causes of interpretative error. There were 3 cases of nonkeratinizing squamous cell carcinoma in biopsies of the oropharynx which were called negative for carcinoma at frozen section. The other 2 cases involved minute foci of squamous cell carcinoma present at margins.

Of the five cases of thyroid tissue vs. parathyroid tissue, three cases showed microfollicular architecture identified as either thyroid or parathyroid at frozen when the reverse was true on permanent section. These three cases required immunohistochemical stains for clarification. Two cases involved scant amounts of parathyroid tissue that were not identified at the edge of the slide.

There were three cases of misinterpreted non-squamous lesions and two cases of metastases that were not appreciated on the slides. One of the misinterpreted non-squamous lesions involved misdiagnosing lymphoma as carcinoma while the others involved confusing follicular variant of papillary thyroid carcinoma for benign thyroid tissue. Of the missed metastases, one was reported as benign thyroid tissue in what was a cystic lymph node involved by papillary thyroid carcinoma. Another case was of a minute fragment of metatypical basal cell carcinoma adjacent to nerve tissue.

**Fig. 3:**
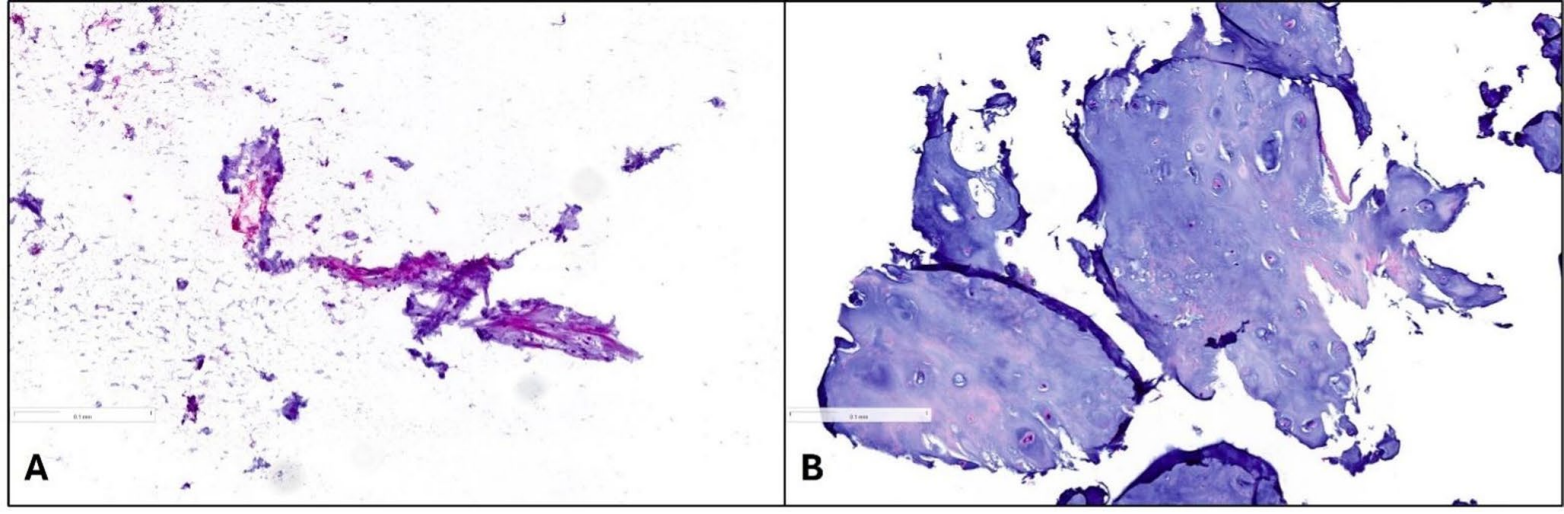
Major technical discrepancy of a cartilaginous neoplasm. **A** (10X) Frozen section reported as “acellular mucinous debris.” **B** (10X) Permanent section of a well-differentiated cartilaginous neoplasm

Major technical discrepancies were found in two cases, including a challenging case of a well-differentiated cartilaginous neoplasm diagnosed as acellular mucinous debris on frozen (Fig. 3). The other technical discrepancy occurred due to a miscommunication about margin inking at time of frozen that resulted in calling back a false positive margin for carcinoma in situ.

## Discussion

The most common means of identifying errors in frozen section interpretation is by comparison of the frozen section results with the permanent section diagnoses. We found interpretative errors to be the largest component of errors in this study. While it is often considered that the primary quality assurance in frozen section analysis is centered on comparison of the frozen section slide diagnosis to final diagnosis, usually based on the evaluation of the permanent section slides, our study revealed other equally important reasons for differences. In addition to interpretation, errors in selecting the appropriate tissue to freeze, particularly for margins from large specimens, insufficiently facing into a frozen tissue block, technical issues, and communication errors all contributed to the diagnostic challenge of accurately communicating a frozen section diagnosis.

Thus, we believe that the entire process of rendering a frozen section diagnosis, including surgeon communication, gross examination and sampling, microtomy, and slide interpretation, is important for process evaluation.

While some of these issues are very difficult to control, vigilance and awareness of the most common causes of errors can serve as a mental checklist in the evaluation of the frozen section.

### Inadequate Sampling of the Gross Specimen

Studies have demonstrated the importance of a specimen-driven approach for margin assessment to mitigate errors [4, 5]. This approach involves excision of the tumor specimen, orientation of the specimen by the surgeon to the pathologist, and targeted gross assessment of the specimen prior to margin sampling [4]. Indeed, the American Joint Committee on Cancer (AJCC) cancer staging manual 8th edition recommends this specimen-driven approach for margin assessment of oral cavity cancers [6]. 

In our study, sampling errors at the gross specimen level often occurred in the evaluation of mucosal margins for squamous dysplasia or carcinoma. These errors appeared to arise from lack of recognition of atypical epithelium and incomplete sampling of long tracts of margins. Careful gross inspection of all frozen specimens by prosectors and pathologists is vital to identifying margin involvement and lesional tissue. In our opinion, the decision of whether to submit en face or perpendicular sections should be made by the pathologist based on the specimen and on which approach they believe will provide the most reliable answer for the surgeon. We recognize that institutional practices may vary and that surgeons may sometimes request margins be performed in a certain manner. Identifying dysplastic squamous epithelium by gross examination is difficult and sometimes impossible. Indeed, surgeons often face similar difficulties in assessing dysplasia on clinical or intraoperative examination.

### Insufficient Sectioning into Frozen Block

Insufficient sectioning of the tissue block at frozen section represented a significant source of error in our study. These types of discrepancies appeared to be fairly evenly spread between different case types (see Table 2). Other groups have examined the value of performing two vs. three levels on routine head and neck frozen sections. Olson et al. showed a slightly reduced discrepancy rate when taking three levels [7]. However, Serinelli et al. showed no difference between routinely performing two or three frozen sections [8]. 

Currently, our primary approach for cutting frozen sections for perpendicular margins and diagnostic tissue (for margins or diagnosis of a larger lesion) is to perform two sections for staining, with one deeper into the block. Having a standardized cutting procedure is useful as a baseline, but multiple factors influence how many sections to cut for staining and how deep to cut. Cutting deeper into the block can be an arbitrary description and the exact amount of tissue taken between levels may vary from person to person and case to case. Our study demonstrates that cutting frozen slides is, in some respects, an act of sampling and, as in any sampling procedure, there are chances of error in cutting depths. In some cases, there was inadequate correlation to the number of tissue pieces in the block compared to the frozen section slide. We would suggest that a specific number of levels in the block are a good starting point, but as the thickness of the tissue sampled varies, it will always be required to observe the frozen block as microtomy continues and matching the tissue on the chuck to the frozen section slide produced. In some of our cases, the amount of tissue remaining in the block should have alerted the microtomist to cut further into the tissue.

The possibility to inadequately cut through to a diagnostic focus also raises the question as to whether all frozen tissue for margins should be consumed by slide production rather than reserving some for permanent section. It has been suggested that examining residual frozen section material provides for quality control of the frozen section process, in part due to what is presumed to be better microtomy and staining of the tissue. However, this also enhances the risk of finding a diagnostic lesion that could have changed the course of surgery. Further, we believe that frozen section slide quality should closely approximate permanent section quality. If significant differences in quality occur, improvement of the frozen section process should be addressed. We do not think cutting through the block takes an appreciably long time and obtaining what we think is the best answer holds the most value. We do this for perpendicular as well as en face margins. If any tumor is seen at any level of a block in a frozen section, our surgeons have considered this as a positive margin. We do note that exhausting the block on lymph node tissues in an effort to find a very tiny metastasis could lead to a false negative that would never be detected. In these cases, we consult with the surgeon to explain the limitations of frozen sections of lymph nodes and proceed with what all feel is best. For some surgeons, we know to consume the lymph node when performing sentinel node evaluation.

There is currently no evidence nor standardized recommendations for sampling additional levels or consuming the block entirely at frozen section. The question of how much tissue to examine at frozen section must be balanced by institutional practices, specimen type, and the intraoperative scenario. For practices such as ours where the standard is to take two levels on most samples, we wonder if sampling could be improved by taking the first level in the “top” half of the tissue after facing completely into the tissue and taking the second level from the “lower” half of the tissue. We do recognize that freezing and cutting tissue is an imperfect process that requires technical experience to develop proficiency and reiterate that tissue sections should be thin (no more than 0.5 cm) [9]. We also recognize the importance of the need to take deeper cuts when faced with ambiguous or nondiagnostic results.

### Interpretative Errors

Interpretive errors accounted for 48% of the major discrepancies and occurred across a range of diagnostic categories: high grade squamous dysplasia/CIS, invasive squamous cell carcinoma, non-squamous neoplasms, parathyroid/thyroid identification, and metastases.

Identifying and grading squamous dysplasia, particularly keratinizing high-grade lesions, is a known diagnostic challenge in head and neck pathology, even on permanent sections. Frozen section artifacts, time constraints, and limited tissue for evaluation present additional challenges during intraoperative consultation.

The cytological and architectural atypia can present in many forms and many of these features can be confined to the basal layers [10]. There is little consensus on how to weigh their cumulative value in grading schemes [11]. As such, the diagnosis of high grade dysplasia suffers from poor inter- and intra-observer reproducibility and can be easily under-recognized or overcalled on frozen section [12]. In addition, reactive changes, radiation-induced changes, metaplasia, and technical artifacts can also all mimic or interfere with recognizing dysplasia. Our data indicated that our pathologists were under-calling high grade dysplasia more frequently than overcalling, but different practices may find the reverse. We encourage frequent review of discrepancy reports, consultation with another pathologist (if possible), and aggressive application of dysplasia criteria to help improve recognition of dysplasia and ensure adequate resection.

As in all specialties, certain diagnostic areas are continually challenging. In our department, errors on frozen section/final diagnosis are reported to the pathologist involved on a regular basis to provide ongoing feedback and education.

Nonkeratinizing variants of squamous cell carcinoma, usually HPV-related, can be particularly challenging to identify, as unlike keratinizing squamous cell carcinoma, nonkeratinizing variants may show more monomorphic cytology. Architecturally, these carcinomas are challenging as they often present as nests or sheets and can blend into the background lymphoid tissue. Errors in these cases often arise from mistaking carcinoma for benign or reactive epithelium or lymphoid tissue [13]. Awareness of the clinical context and tumor patterns can aid in detection [14, 15]. 

Another source of diagnostic error was the failure to recognize non-squamous malignancies. Our study revealed one case of large cell lymphoma misclassified as carcinoma. Melanoma and undifferentiated sarcomas can also mimic epithelial neoplasms. In small biopsies or lymph node specimens, broadening the diagnosis to include hematolymphoid or melanocytic neoplasms by reporting “malignant cells present,” for example, can communicate the pertinent findings while acknowledging the diagnostic limits of frozen section in a particular case.

Discrepant non-squamous neoplasms also included two cases of papillary thyroid carcinoma that were misclassified on frozen sections of thyroid. One of these cases was a follicular variant of PTC and the other was a microcarcinoma. Freezing of thyroid lesions for intraoperative diagnosis is discouraged, particularly in cases of papillary thyroid carcinoma which are notoriously susceptible to freezing artifact and often won’t show similar nuclear features as may be seen on permanent section [16]. Psammoma bodies, papillary architecture, and tightly packed groups of cells with considerable nuclear overlap can be a “tip-off,” but may not be present. Capsular lesions, such as follicular adenomas or follicular carcinomas, can require submission of the entire capsule for definitive diagnosis, which is not a task suited to frozen section analysis [12, 17]. It is recommended to discuss these cases with the surgeon to defer the diagnosis to permanent sections if possible.

Intraoperative frozen sections are routinely employed to confirm the presence of parathyroid tissue as opposed to fatty tissue, thymus, thyroid, or lymph node tissue. This is an extremely reliable process but it also presents diagnostic challenges [18]. Differentiating between parathyroid and thyroid tissue can be particularly difficult when the parathyroid tissue shows follicular architecture or when thyroid tissue contains stromal fat [19]. Exacerbating this difficulty is the fact that parathyroid glands can be found within the thyroid and thyroid tissue can be ectopic to the soft tissue of the neck. Features that favor parathyroid tissue include solid or nested features with a mixed population of chief and oxyphilic cells. These cells are usually more monotonous with smaller, darker nuclei than thyroid follicular cells, but these distinctions can be difficult. The presence of fat or a rim of normal parathyroid gland can also be helpful to support parathyroid origin. Use of a polarizer in these cases to detect birefringent calcium crystals in the colloid of thyroid follicular epithelium favors the presence of thyroid tissue. However, the absence of calcium crystals does not exclude thyroid tissue [17]. 

Metastatic deposits can be especially easy to overlook. Anchor bias or a busy workload can lead to missing relevant findings. Our two cases of major discrepancies exemplified this. One of these cases was of an overlooked minute focus of tumor present in a nerve biopsy that was part of a larger case with multiple other frozen sections, many of which arrived in the laboratory concurrently. Our other case was of a lymph node with metastatic papillary thyroid carcinoma that was reported as normal thyroid tissue. This specimen was labeled as “Thyroid nodule vs parathyroid, please assess by frozen and weight.” Caution and adequate review of the entire gross and microscopic tissue may aid in avoiding such errors.

We have not attempted to utilize immunohistochemical staining as an adjunct to frozen section evaluation at this time. We believe this technique to possibly hold the most value for evaluation of lymph node metastases or detecting very small foci of perineural invasion.

## Conclusion

Major frozen section errors in head and neck cases occur relatively infrequently. However, these errors can significantly impact intraoperative clinical decisions and patient outcomes.

Gross sampling of large specimens, insufficient cutting of the frozen tissue block and interpretation factors are the major sources of errors in frozen section discrepancies. A potential limitation of our study is that we did not re-review cases coded as ‘agree’ or ‘no discrepancy’, which could underestimate the number of discrepancies. In this study, errors in selecting the current tissue to examine by frozen section and insufficient cutting of the tissue block accounted for 50% of the discrepancies. In diagnoses related to the very common problem of determining squamous dysplasia/carcinoma in situ in margins, all three of these issues had an impact. Further we believe that the possibility to inadequately cut through to a diagnostic focus in a block also raises the question as to whether all frozen tissue for margins should be consumed by slide production, rather than reserving some for permanent section.

Steps to reduce these discrepancies may include regular review of specimen-specific sampling protocols, seeking continuous education on diagnostic criteria, and asking for help in difficult cases. In addition, institutions may benefit from implementing frozen section sign-out safety nets such as second-review policies for high-risk cases and periodic frozen section quality assurance conferences [20]. 

## Data Availability

The generated data are not publicly accessible. Original files are available from the corresponding author upon reasonable request.
